# The microclimatic effects of the native shrub *Ephedra californica* (Mormon tea) in California drylands

**DOI:** 10.3389/fpls.2024.1396004

**Published:** 2024-10-09

**Authors:** Nargol Ghazian, Rachel King, Mario Zuliani, Christopher J. Lortie

**Affiliations:** ^1^ Department of Biology, York University, Toronto, ON, Canada; ^2^ National Center for Ecological Analysis and Synthesis (NCEAS), Santa Barbara, CA, United States

**Keywords:** facilitation, foundation shrub, microclimate, heterogeneity, richness, abundance

## Abstract

**Introduction:**

The impacts of climate change can be profound in many ecosystems worldwide, including drylands such as arid and semi-arid scrublands and grasslands. Foundation plants such as shrubs can provide microclimatic refuges for a variety of taxa. These shrubs can directly influence micro6 environmental measures, and indirectly increase the local environmental heterogeneity as a result. We examined the hypothesis that, in comparison to an open gap, foundation shrubs improve the microclimate beneath their canopy and that microclimate is in turn a significant predictor of annual vegetation. The following predictions were made: 1) mean air temperature (NSAT), ground temperature (SGT), and vapour pressure deficit (VPD) will be significantly lower under the shrubs than in the open microsites; 2) shrub canopy size predicts microclimate; 3) site-level aridity estimates and percent shrub cover influence annual plant abundance and richness; and 4) the site13 level mean of NSAT and VPD predict annual plant abundance and richness.

**Methods:**

Our study took place in Southwestern California, U.S.A. We used a handheld device with a probe to measure microclimatic variables such as near-surface air temperature (NSAT), near-surface relative humidity (NSRH), and surface ground temperature (SGT) at the shrub species *Ephedra californica* and in the open gap, across six sites in California, United States. Air temperature and RH were then used to calculate VPD. The mean number of vascular plant species across each site was also recorded.

**Results & discussion:**

Only SGT was significantly reduced under shrub canopies. Canopy volume was not a significant predictor of all three microclimatic variables, demonstrating that even small, low-stature shrubs can have facilitative effects. Furthermore, total shrub cover and aridity at sites significantly predicted mean plant richness and abundance. There were significantly more plants associated with shrubs and there were significantly more species associated with the open. Mean air temperature and VPD at the site-level significantly predicted vegetation abundance and richness, though microsite-level differences were only significant for richness. Foundation shrubs are a focal point of resiliency in dryland ecosystems. Understanding their impact on microclimate can inform us of better management, conservation, and restoration frameworks.

## Introduction

Facilitation, defined as an interaction where one or more species (beneficiary) benefit from another species (benefactor) while none are harmed (Bertness and Leonard, 1997), is well-studied in community ecology (Brooker et al., 2007; McIntire and Fajardo, 2014). This interaction is often present in relatively high-stress environments, such as drylands, and generally, the magnitude of this interaction also increases with an increase in abiotic pressures (Bertness and Callaway, 1994). Foundation species like shrubs can act as structural agents of facilitation through many mechanistic pathways, one of which is microclimatic amelioration (Filazzola et al., 2017). Shrub understories provide more favorable environments for plants and animals by buffering against extreme temperatures, offering refuge from direct solar radiation, and increasing soil moisture (Filazzola and Lortie, 2014; Flores and Jurado, 2003; Holzapfel and Mahall, 1999). Thus, foundation shrubs are critical for community structure and diversity in many dryland ecosystems (Ivey et al., 2020; Zuliani et al., 2021).

Foundation shrubs are key components of environmental heterogeneity. Foundation shrubs physically ameliorate the understory microclimate compared to the open gap (Anthelme et al., 2014; Filazzola et al., 2017; Zuliani et al., 2021); thus, resulting in environmental heterogeneity as a byproduct of this microclimatic difference. Environmental heterogeneity (EH), defined as non-uniformities in physical and ecological landscape characteristics (Dronova, 2017), can be divided into many subcomponents including climatic, vegetation, land cover, soil, and topography heterogeneity (Stein and Kreft, 2015). ‘Microclimate’ is the climate experienced in the lower 2m of the atmosphere and the upper 0.5-1m of the soil and is dependent on local topography, soil type, and vegetation (Stoutjesdijk and Barkman, 2014). Foundation plans provide resilience and microclimatic heterogeneity in drylands (Lortie et al., 2022), where elevated mean temperatures, fluctuations in temperature and precipitation, and prolonged drought episodes are on the rise (Maestre et al., 2021). Resiliency is a concept that refers to the ability of a system to resist changes in response to perturbations (Van Meerbeek et al., 2021). Plants can buffer perturbations that are the result of harsh climatic conditions (De Frenne et al., 2019). The impacts of plants in buffering climate have been primarily tested through landscape-level analyses, such as NDVI (Normalized Difference Vegetation Index) or vegetation land cover (Bagley et al., 2017); however, the structure of foundation species offers significant ecological functions that are crucial for offering climatic refuge at finer scales (Milling et al., 2018).

Like plant canopies in other systems, shrub canopies can, directly and indirectly, buffer climatic regimes. Plant canopies offer mediation from harsh climates by increasing environmental heterogeneity and cover (De Frenne et al., 2019). The quantity, quality, and temporal distribution of incoming sunlight are governed by the canopy’s structure, which also affects air movement, which in turn affects temperature and precipitation regimes via boundary layer effects (Jennings et al., 1998). Shrub volume is directly associated with micro-environmental conditions, with larger shrubs showing a greater environmental heterogeneity in their understory (Alday et al., 2014). Similar to other studies with plants (Yang et al., 2017), this is likely due to the greater heterogeneity of conditions beneath larger plant canopies. Many dryland organisms are vulnerable to small, fine-scale oscillations in addition to large-scale changes (Shrode and Gerking 1977; Hadley 1970), which can further push species past the point of no return. Thus, foundation shrubs, including *Ephedra californica*, can help facilitate some of these species (Lortie et al., 2018) through small-scale climatic amelioration. The idea of vegetation and microclimate in arid systems is well-explored (El-Bana et al., 2002; Jankju, 2013; Xue et al., 2019) but not necessarily concerning the size of the foundation species at multiple arid sites. Shrub volume measured and reported to such an extent at multiple arid sites in addition to its impact on annual plant richness and abundance is where the novelty of this study lies.

*Ephedra Californica* (Mormon Tea) is a foundation shrub native to the Southwestern regions of California that can occur dominantly or co-dominantly with *Larrea tridentata* (Sawyer et al., 2009), a flowering shrub that is often found in sandy soils, desert pavements, and the well-developed cryptogram layer of the Mojave (Braun et al., 2021; Sawyer et al., 2009). In this study, we measured microclimatic variables, including near-surface air temperature (NSAT), near-surface relative humidity (NSRH), and surface ground temperature (SGT) using a handheld device across six sites in California. NSAT is a point-station, air temperature measure that differs from a direct land surface measure of temperature and is a critical factor in the process of energy exchange between the land and the atmosphere (Xu et al., 2012). SGT (also referred to as Land Surface Temperature in the literature) is the measure of temperature directly on land as opposed to NSAT (Good et al., 2017). Similar to NSAT, NSRH refers to the measurement of the air moisture content near the surface (Pfahl and Niedermann, 2011). We used NSAT and NSRH to calculate vapor pressure deficit (VPD). We tested the hypothesis that foundation shrubs ameliorate the microclimate underneath their canopy relative to the open gap and that microclimate is a significant predictor of annual vegetation. The following predictions were made: 1) mean air temperature (NSAT), ground temperature (SGT), and vapor pressure deficit (VPD) will be significantly lower under the shrubs than in the open microsites; 2) shrub canopy size predicts microclimate; 3) site-level aridity estimates and percent shrub cover influence annual plant abundance and richness; and 4) the site-level mean of NSAT and VPD predict annual plant abundance and richness. Shrubs and vegetation improve microclimate and can thus provide key environmental heterogeneity that is crucial to the persistence of many dryland species given the current trajectory of climate change.

## Materials & methods

### Site description

We surveyed a total of six sites across arid and semi-arid areas during the winter of 2023 (**Table 1**). The sites were located in Southwestern California, United States, and divided into two main ecological areas: Cuyama Valley and Carrizo Plain National Monument. The surveys took place between February 13 and 23, 2023. We surveyed a total of three sites in Cuyama Valley and three sites in the Carrizo Plain. Sites were always surveyed in the morning at approximately 9:00 AM. The year this study took place was considered a high precipitation year, characterized by a ‘superbloom’ and winter rainfall exceeding the long-term means by ≥70% (Jennings and Berry, 2023). We chose this study period because the early species of annuals normally begin germinating in March (Jennings and Berry, 2023), but because of the superbloom and high rainfall periods, they germinated earlier in late January (Jennings, 2001). We retrieved daily min and maximum temperatures (F) and precipitation (in) for each day of the study period from the National Oceanic and Atmospheric Administration (NOAA) (https://www.noaa.gov/; **Supplementary Appendix C**) using the nearest satellite located in New Cuyama, Cuyama Valley. Moreover, site-level mean annual temperature (MAP) and mean annual precipitation (MAP) were obtained from WorldClim (https://www.worldclim.org/data/index.html) at a 1km resolution. We further calculated DeMartonne’s aridity values using the following formula: aridity = *P*/(*T* + 10) where *P* = annual precipitation and *T* = mean annual temperature and classified the climate gradient according to Croitoru et al. (2013).

**Table 1:** Sites. List of study sites in California, United States surveyed for this study and their respective geographical coordinates.

*Site Code*	*Semi-arid* *Region*	*Latitude*	*Longitude*	*MAT* *(°C)*	*MAP* *(mm)*	*Aridity*	*Regional * *Gradient Classification*
***Cuyama_1***	San Joaquin	34.849	-119.483	14	136	5.67	Arid
***Cuyama_2***	San Joaquin	34.854	-119.486	14.1	137	5.69	Arid
***Cuyama_3***	San Joaquin	34.938	-119.481	14.2	139	5.74	Arid
***Carrizo_3***	San Joaquin	35.163	-119.675	14.5	149	6.08	Arid
***Carrizo_4***	San Joaquin	35.116	-119.621	14.7	147	5.95	Arid
***Carrizo_soda_shrub***	San Joaquin	35.119	-119.629	14.7	148	5.99	Arid

Mean annual temperature (MAT) and mean annual precipitation (MAP) were extracted from WorldClim and used to calculate DeMartonne’s aridity index.

### Vegetation sampling & microclimate

We selected 30 pairs of shrub-open microsites at each study site. *Ephedra californica* was the dominant shrub across all these sites and there were no other dominant or co-dominant shrub species present. The length (at longest axis), width (perpendicular to length), and height to the tallest vegetation branch that had green tissue (Mormon tea is a shrub with many branches) were measured for every shrub (Lortie et al., 2018). We used the equation for the volume of a hemisphere ( $v=\frac{2}{3}\;\text{π}r^{3}$ ) to calculate canopy volume and the equation for the area of a circle ( $a=\;\text{π}r^{2}$ ) to calculate the canopy area (Liczner et al., 2016; Lortie et al., 2018). We recorded the percent shrub cover at each site within a 20m radius at the center of the site via *Google Earth* composite satellite images at a spatial resolution of 30cm (Hestrio et al., 2021; Owen et al., 2024). Within each circular plot (20m in radius), the length at the longest axis of every shrub was measured and used to calculate the individual shrub cover area using a formula of a circle (Liczner et al., 2016; Lortie et al., 2018). Shrub covers were then summed and divided by the total area of the circular plot to produce percent shrub cover estimates of each site (**Supplementary Appendix D, L**).

We used the Mengshen Digital Temperature and Humidity Meter (Mengshen, 2023) to record near-surface air temperature (NSAT, °C) and near-surface relative humidity (NSRH, %). To record these microclimatic variables, the handheld probe was positioned at approximately ∼10-15cm above the soil at the north side of the shrub, immediately within the canopy dripline (inside), and 1m away from a shrub in the adjacent, interstitial open area (outside). We used a cover to stop direct sunlight on the probe bulbs when air temperature and RH were being recorded. Furthermore, we also used the Etekcity Laser Thermometer Gun (Etekcity, 2023) to record surface ground temperature (SGT, °C) both underneath the shrub canopy at the north side of the shrub, and in the open area located adjacently 1m away from a shrub. We then used air temperature and RH to calculate the vapor pressure deficit (VPD) as vegetation structure and microtopography, directly and indirectly, impact this measurement (Novick et al., 2024). We used the following equation: $VPD=SVP*\left(1-\frac{RH}{100}\right)$ where saturation vapor pressure (SVP) is the maximum amount of water vapor that air can hold as a certain recorded temperature and RH = relative humidity.

To further explore if microclimate changes depending on the site-level differences in vegetation, we conducted vegetation surveys in shrub and open microsites at each study site. We identified all vascular plant species present in four paired 1 m^2^ quadrats at each microsite (8 total per site). In shrub microsites, one of the paired quadrats was placed under the drip line of randomly selected shrubs on the south-facing side and the paired open quadrat was placed due south with 1m spacing between the two quadrats. In open microsites, each pair of quadrats was randomly placed in what we deemed as the center of the site. We summarized the results of the eight plots to determine the mean annual plant abundance and richness.

### Statistical analyses

All statistical analyses were done using R version 4.3.1 (R Core Team, 2024). Data and codes are publicly available on Zenodo (Ghazian and Lortie, 2023). Data distribution was examined using Q-Q plots, and homoscedasticity and normality were tested (Schützenmeister et al., 2012). The relationship between NSAT, SGT, NSRH, and VPD was examined using Pearson’s correlation (Benesty et al., 2009). The relationship between canopy area and volume was also examined using Pearson’s correlation, as well. We fit Generalized Linear Models (GLM) to test for differences in shrub volume and its effects on air temperature, VPD, and ground temperature for the open versus the shrub. GLM dispersion parameters with AIC scores were used to compare and select the appropriate family to fit to models (Richards et al., 2011). *Post-hoc* tests were done using the function *emmeans* from the emmeans R package (Lenth and Herve, 2019). We then used Generalized Linear Mixed Models (GLMM) to model shrub volume as a predictor of each microclimatic variable, with microsite nested in site to serve as a covariate. We further explored if mean plant richness and abundance were predicted by percent shrub cover, aridity, mean NSAT, and mean VPD using GLMs. We included predictors in these models by testing multicollinearity using the *Performance* package and excluded those that lead to high variance inflation factors (VIF) (Daoud, 2017), and hence high collinearity (**Supplementary Appendix A, B**).

## Results

**Figure 1:**
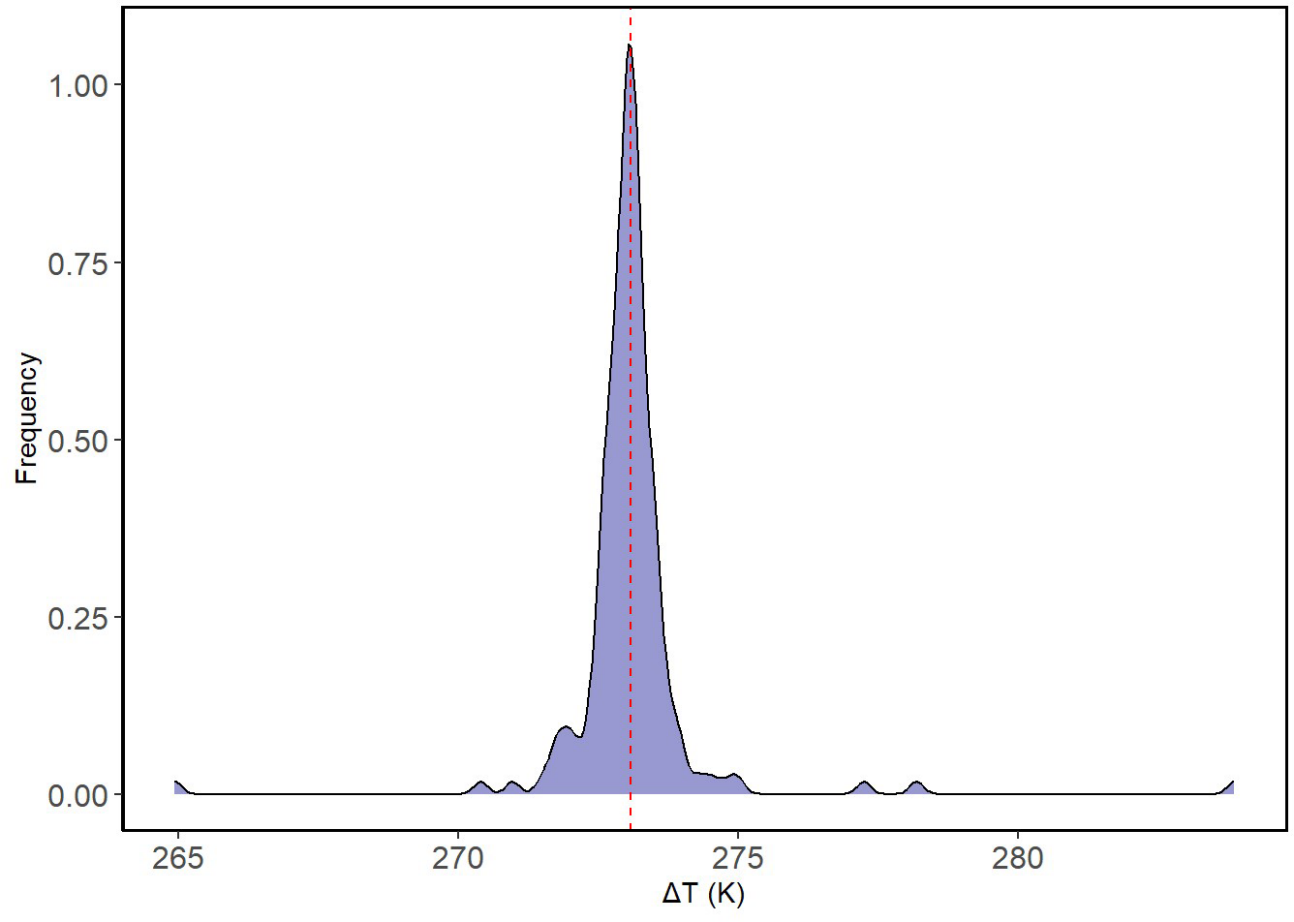
Frequency distribution. The frequency distribution of temperature differences between the open and shrub is presented. The x-axis represents the difference in temperature in Kelvin while the y-axis represents the particular frequency ΔT that was observed. Dashed red line is the mean of temperature changes.

**Figure 2:**
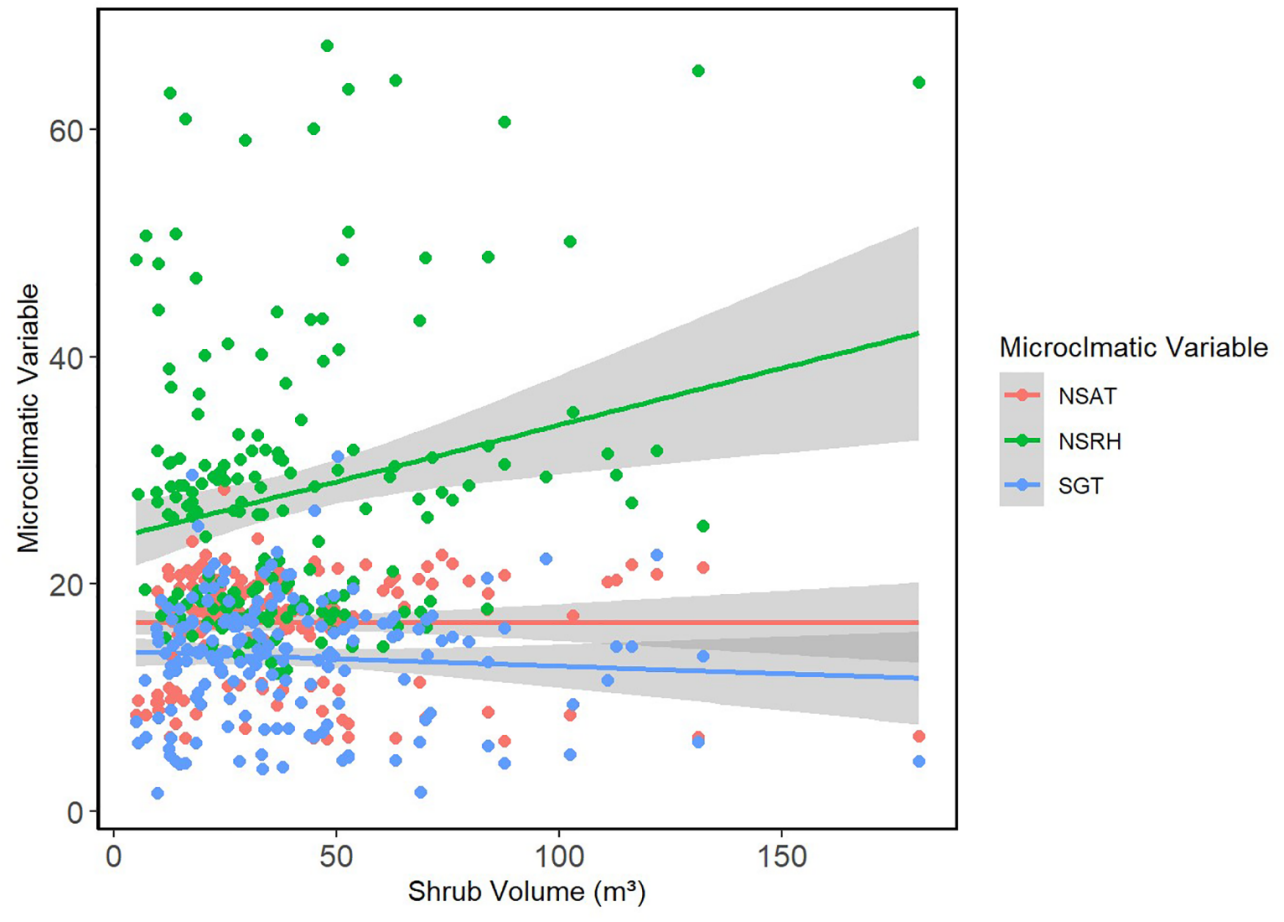
Shrub volume vs. microclimatic measures. The relationship between shrub volume (m^3^) and microclimatic measures including NSAT (°C), NSRH (%), and SGT (°C) are presented using points. Smoothed means are fitted using the linear method. Colors represent the different key microclimatic variables.

A total of 180 shrub-open microsites were surveyed for microclimatic measures (n = 180). Mean shrub volume and area for *E. californica* were 39.64 ± 27.80 m^3^ and 37.16 ± 22.06 m^2^, respectively (**Supplementary Appendix F**). Shrub area and canopy volume were significantly, and positively related (Pearson’s product-moment correlation = 0.92, p<0.01; **Supplementary Appendix G**); thus, we used shrub volume as a proxy for size in all our models. Percent shrub cover at study sites ranged from 6.68-31.18% (**Supplementary Appendix D**). **Table 2** provides the mean air temperature and VPD under the shrub and in the open at each site. The lowest mean air temperature was recorded in the open at Carrizo_soda_shrub (15.06 ± 3.48 °C) and the lowest mean VPD was recorded under the shrub (0.55 ± 0.16 kPa) in Cuyama_3. **Figure 1** depicts the frequency distribution of change in air temperature between the open and the shrub in Kelvin. Mean ground temperature was the only microclimatic parameter significantly lower under the shrub than in the open (Estimated Marginalized Mean (EMM) 13.5 ± 0.39 °C, p<0.01; **Figure 2**).

**Table 2:** Air temperature & VPD summary.

*Site Code*	*Semi-arid Region*	*Microsite*	*Mean ± SD* *NSAT* *(°C)*	*Mean ± SD* *VPD* *(kPa)*
***Cuyama_1***	San Joaquin	open	29.14 ± 2.59	1.67 ± 0.41
***Cuyama_1***	San Joaquin	shrub	19.73 ± 1.98	1.59 ± 0.25
***Cuyama_2***	San Joaquin	open	20.78 ± 1.32	1.76 ± 0.22
***Cuyama_2***	San Joaquin	shrub	21.08 ± 1.69	1.81 ± 0.23
***Cuyama_3***	San Joaquin	open	8.46 ± 1.91	0.56 ± 0.18
***Cuyama_3***	San Joaquin	shrub	8.45 ± 1.97	0.55 ± 0.16
***Carrizo_3***	San Joaquin	open	17.24 ± 1.21	1.66 ± 0.16
***Carrizo_3***	San Joaquin	shrub	17.62 ± 1.40	1.70 ± 0.19
***Carrizo_4***	San Joaquin	open	17.34 ± 0.54	1.62 ± 0.08
***Carrizo_4***	San Joaquin	shrub	17.33 ± 1.10	1.61 ± 0.13
***Carrizo_soda_shrub***	San Joaquin	open	15.06 ± 3.48	1.35 ± 0.34
***Carrizo_soda_shrub***	San Joaquin	shrub	15.12 ± 3.74	1.39 ± 0.38

Mean near-surface air temperature (NSAT) and vapor pressure deficit (VPD) with their standard deviations are provided across each site, under the shrub, and in the open.

Overall, shrub volume did not have a significant effect on air temperature in fixed-effect models (GLMM estimated β = 0; **Table 3**). The same was observed for VPD (GLMM estimated (β = 0) and ground temperature (GLMM estimated β = -0.01). The confidence intervals for all three estimates were also narrow, which further confirms the weak effects of shrub volume on these microclimatic variables.

**Table 3:** Model summary I.

*Predictors*		*Estimates*	*CI*	*p-value*	*Standard Error* *(SE)*
***NSAT (°C)***	**(Intercept)**	16.44	12.82-20.07	**0.01**	1.85
	**shrub_volume**	0	-0.01-0.02	0.57	0.0059
	Number of Observations: 180 Marginal R^2^: 0 N_sites_: 6				
***VPD (kPa)***	**(Intercept)**	1.43	1.06-1.80	**0.01**	0.19
	**shrub_volume**	0	-0.01-0.04	0.59	0.001
	Number of Observations: 180 Marginal R^2^: 0 N_sites_: 6				
***SGT (°C)***	**(Intercept)**	14.09	10.5-17.67	**0.01**	1.83
	**shrub_volume**	-0.01	-0.03-0.01	0.17	0.01
	Number of Observations: 180 Marginal R^2^: 0.005 N_sites_: 6				

Contrast of microclimatic measurements estimated using Generalized Linear Mixed Models (GLMM). Microsite was nested within the site code. Significant p-values are in bold. CI represents the 95% confidence interval.

**Figure 3:**
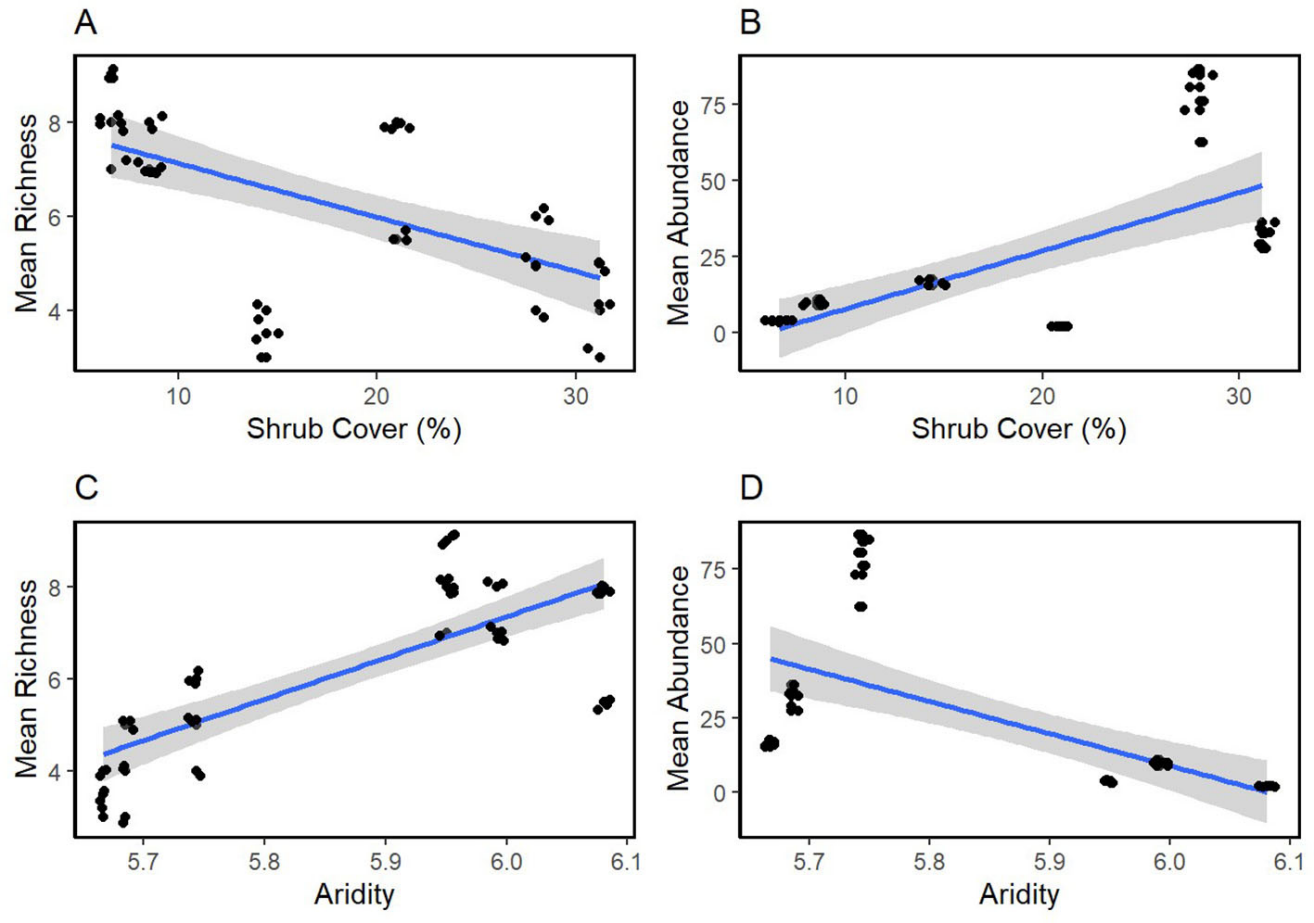
Vegetation measures (I) Mean plant abundance and richness regressed against percent shrub cover (%) **(A, B)** and aridity **(C, D)**. Smoothed means are fitted using the linear method.

**Figure 4:**
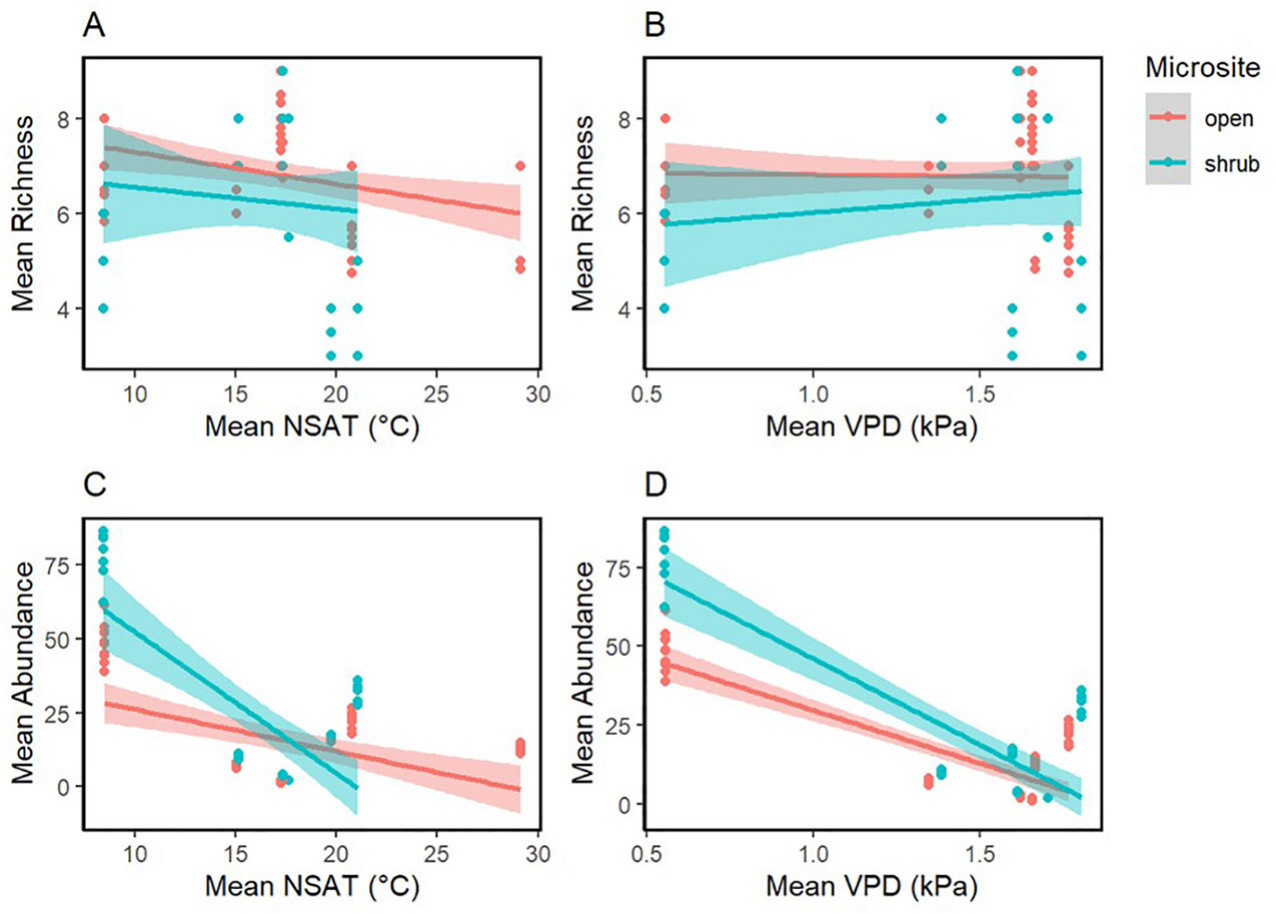
Vegetation measures II. Mean plant abundance and richness regressed against mean NSAT (°C) **(A, C)** and mean VPD (kPa) **(B, D)**. Smoothed means are fitted using the linear method. Color represents the microsite.

A total of 27 different annual plant species were found under shrubs and in the open (**Supplementary Appendix E**). *Bromus madritensis rubens* (red brome) was the most abundant species observed under the shrubs, while *Lasthenia gracilis* (needle goldfield) was the most abundant species in the open. *Astragalus lentiginosus nigricalycis* (spotted locoweed), *Brassica nigra* (black mustard), *Lactuca serriola* (prickly lettuce), *Lupinus microcarpus* (chick lupine) were the equally the least abundant species under the shrub. *Lupinus microcarpus* (chick lupine) was also the least abundant species in the open. There were four species exclusively found under shrubs, including *Brassica nigra* (black mustard), *Eremalche exilis* (white mallow), *Lactuca serriola* (prickly lettuce), *Pholistoma membranaceum* (white fiesta flower). Both site-level shrub cover and aridity were significant predictors of plant abundance and richness (**Figure 3** and **Table 4**). Mean plant abundance was significantly greater under the shrubs (EMM 23 ± 1.82, p<0.01), while mean richness was significantly greater in the open (EMM 6.82 ± 0.16, p<0.01). Site-level mean air temperature and mean VPD were significant predictors of plant richness both under the shrub and in the open, but not abundance (**Figure 4**, **Table 5**).

**Table 4:** Model summary II.

	*Predictors*	*df*	*Deviance Residuals*	*df residuals*	*Residual* *Deviation*	*p-value*
***Mean richness***	**NULL**			107	227.87	
	**shrub_cover**	1	35.57	106	192.30	**0.01**
	**microsite**	1	8.89	105	183.4	**0.01**
	**aridity**	1	66.72	104	117.13	**0.01**
***Mean*** ***abundance***	**NULL**			107	51383	
	**shrub_cover**	1	20589.9	106	30793	**0.01**
	**microsite**	1	1633.3	105	29160	**0.01**
	**aridity**	1	6803.6	104	22356	**0.01**

Analysis mean annual plant richness and abundance are shown using a Generalized Linear Model (GLM). Significant p-values are bolded. Given model has the lowest AIC for the family of fit.

**Table 5:** Model summary III.

	*Predictors*	*df*	*Deviance Residuals*	*df residuals*	*Residual Deviation*	*p-value*
***Mean richness***	**NULL**			107	227.87	
	**mean_NSAT**	1	9.75	106	218.12	**0.01**
	**microsite**	1	9.15	105	208.96	**0.01**
	**mean_VPD**	1	38.31	104	170.65	**0.01**
***Mean*** ***abundance***	**NULL**			107	51383	
	**mean_NSAT**	1	14719.1	106	36664	**0.01**
	**microsite**	1	419.5	105	36244	0.08
	**mean_VPD**	1	21743.3	104	14501	**0.01**

Analysis mean annual plant richness and abundance are shown using a Generalized Linear Model (GLM). Significant p-values are bolded. Given model has the lowest AIC for the family of fit.

## Discussion

Abiotic amelioration via foundation shrubs is a key component of many dryland ecosystems. Herein, we examined the microclimate of the foundation shrub species, *Ephedra californica* in order to determine the relative significance of shrub volume on microclimate and the role of microclimate in predicting vegetation richness and abundance. These were tested across six sites in the drylands of California. We hypothesized that, in comparison to an open gap, foundation shrubs improve the microclimate beneath their canopy and that microclimate is in turn a significant predictor of annual vegetation. Despite our prediction that all microclimatic variables under the shrub would be significantly lower than in the open, we only observed significantly lower ground temperature under the shrub. Shrub volume was not a significant predictor of air temperature, ground temperature, and VPD. Thus, we propose that *E. californica* is a good benefactor species when it comes to facilitating some micro-environmental measures compared to the open gap; though, its role in microclimatic amelioration is weakly dependent on size. Furthermore, we concluded that site-level percent shrub cover and aridity are significant predictors of vascular plant abundance and richness. Mean air temperature and VPD at the site level significantly predicted vegetation richness and abundance, however, microsite-level differences were only significant to plant richness. Mean plant abundance was greater under shrubs suggesting that shrub cover has an impact on microclimate, and the environmental heterogeneity induced by shrubs that may act on different spatial scales, hence altering the dynamics of distribution and establishment of the herbaceous plant community (Madrigal et al., 2008). We suggest that one factor may be microclimatic amelioration, such as cooler ground temperatures, by foundation species that allow for the presence of species that would otherwise be absent. This is consistent with the results of Soliveres and Maestre (2014) who observed 29% more species associated with shrubs in drylands compared to the open. Furthermore, we observed that mean plant richness was higher in the open. We believe that this may be due to the fact some shrubs restrict the establishment and survivorship of certain annual plants because the reduction of light by shrub canopy can limit the photosynthetic activity of annuals (Swank and Oechel, 1991). Semi-arid annual plants attain high net photosynthetic rates by having very high light saturation levels (Werk et al., 1983). A reduction in radiation by shrubs can reduce resource acquisition (Jackson and Caldwell, 1992). Thus, we suggest that plant-plant interactions and their responses to ongoing climate change are more complex than previously thought (Seiferling et al., 2014), but these interactions are nonetheless crucial in maintaining biodiversity in dryland ecosystems, particularly given the number of species that exclusively associate with shrubs. Though beyond the scope of our study, we suggest that future experiments consider plant traits as a tool to understand why some species are more benefited than others.

More than 30% of the total land area in California is defined as a semi-arid ecoregion (Syphard et al., 2022). Shrubs create heterogeneity in these regions by creating variation in physical structure and thus provide a generalized facilitation function of ameliorating microclimate (Braun et al., 2021). Shrub canopies create micro-environmental benefits including increasing shade, reducing radiation load, increasing night-time winter temperature, and increasing soil moisture, and canopy structure play an important role in differences observed in these micro-environmental parameters (Jankju, 2013). In this study, we observed the facilitative effects of *E. californica* are not dependent on size. This shows that even the canopy of smaller, low-stature, long-lived ancient shrubs can nonetheless provide fine-scale climatic refuges for other species (Ivey et al., 2020; Lortie et al., 2018, 2020). Maintaining the biodiversity of semi-arid regions is crucial as it translates to the overall biodiversity of the entire region of California. There is a direct relationship between environmental heterogeneity (EH) and biodiversity, with species richness increasing with greater EH (Agra et al., 2021). Maintaining *E. californica* presence and promoting its persistence in the region through conservation, restoration, and management will result in greater heterogeneity in the region and thus higher regional biodiversity. Shrubs and annual plants enable small areas in the species niche that can allow species persistence in a shifting climate (Brooker et al., 2018). Thus providing animal species with the option to move around and behaviorally mitigate climate change effects. Hence, management and restoration efforts need to focus on not only protecting existing shrub species but also restoring and managing disturbed scrubland and they are key to the persistence of biodiversity. Supporting the persistence of other taxa through foundation shrubs like *E. californica* that both directly and indirectly influence microclimatic heterogeneity is crucial in the future well-being of dryland ecosystems under the current climate change paradigms.

## Data Availability

The datasets presented in this study can be found in online repositories. The names of the repository/repositories and accession number(s) can be found in the article/**Supplementary Material**.
