# Supplementary material for: The microclimatic effects of the native shrub Ephedra californica (Mormon tea) in California drylands
Source: Front Plant Sci. 2024 Oct 9;15:1396004. doi: 10.3389/fpls.2024.1396004 (PMC11496180; doi:10.3389/fpls.2024.1396004)
Supplement: Supplementary file 1 [file DataSheet1.docx]

**Supplementary Appendix**

1. **Multicollinearity** The variance inflation factors (VIF) for the GLMs where site level aridity and percent shrub cover are predictors of annual vegetation. VIFs <5 indicate low collinearity. CI is the 95% confidence interval.

| **Measure** | **Term** | **VIF** | **CI** | **Standard Error** | **Tolerance** | **Tolerance CI** |
| --- | --- | --- | --- | --- | --- | --- |
| **Mean richness** | shrub_cover | 2.93 | 2.26-3.96 | 1.71 | 0.34 | 0.25-0.44 |
|  | microsite | 1.01 | 1-4.84*10^8^ | 1.00 | 0.99 | 0-1 |
|  | aridity | 2.93 | 2.26-3.96 | 1.71 | 0.34 | 0.25-0.44 |
| **Mean abundance** | shrub_cover | 2.93 | 2.26-3.96 | 1.71 | 0.34 | 0.25-0.44 |
|  | microsite | 1.01 | 1-4.84*10^8^ | 1.00 | 0.99 | 0-1 |
|  | aridity | 2.93 | 2.26-3.96 | 1.71 | 0.34 | 0.25-0.44 |
|  |  |  |  |  |  |  |
|  |  |  |  |  |  |  |

1. **Multicollinearity** The variance inflation factors (VIF) for the GLMs where mean near-surface air temperature (NSAT) and vapour pressure deficit (VPD) are predictors of annual vegetation. VIFs <5 indicate low collinearity. CI is the 95% confidence interval.

| **Measure** | **Term** | **VIF** | **CI** | **Standard Error** | **Tolerance** | **Tolerance CI** |
| --- | --- | --- | --- | --- | --- | --- |
| **Mean richness** | mean_NSAT | 3.06 | 2.33-4.21 | 1.75 | 0.33 | 0.24-0.43 |
|  | microsite | 1.05 | 1-3.94 | 1.02 | 0.96 | 0.25-1 |
|  | mean_VPD | 3.03 | 2.31-4.16 | 1.75 | 0.33 | 0.24-0.43 |
| **Mean abundance** | mean_NSAT | 3.06 | 2.33-4.21 | 1.75 | 0.33 | 0.24-0.43 |
|  | microsite | 1.05 | 1-3.94 | 1.02 | 0.96 | 0.25-1 |
|  | mean_VPD | 3.03 | 2.31-4.16 | 1.75 | 0.33 | 0.24-0.43 |
|  |  |  |  |  |  |  |
|  |  |  |  |  |  |  |

1. **Weather Data** Daily maxima and minima for temperatures (F), as well as total precipitation (inches), were obtained from the National Oceanic and Atmospheric Association (NOAA) for the February 13^th^- 23^rd^, 2023 study period. The satellite station nearest to all six study sites was located in Cuyama Valley, New Cuyama, CA.

| **Year** | **Month** | **Day** | **Max Temperature**  **(F)** | **Min Temperature**  **(F)** | **Mean**  **Temperature**  **(F)** | **Precipitation (in)** |
| --- | --- | --- | --- | --- | --- | --- |
| 2023 | 02 | 13 | 60 | 37 | 48.6 | 0 |
| 2023 | 02 | 14 | 47 | 23 | 46.5 | 0 |
| 2023 | 02 | 15 | 48 | 20 | 34 | 0 |
| 2023 | 02 | 16 | 55 | 26 | 40.5 | 0 |
| 2023 | 02 | 17 | 56 | 24 | 40 | 0 |
| 2023 | 02 | 18 | 57 | 26 | 41.5 | 0 |
| 2023 | 02 | 19 | 58 | 28 | 43 | 0 |
| 2023 | 02 | 20 | 67 | 31 | 49 | 0 |
| 2023 | 02 | 21 | 64 | 29 | 46.5 | 0.01 |
| 2023 | 02 | 22 | 44 | 26 | 35 | 0.01 |
| 2023 | 02 | 23 | 45 | 31 | 38 | 0.01 |

1. **Shrub Cover** Percent shrub cover within a 20m radius was estimated at the centre of each site using *Google Earth* composite satellite images at a spatial resolution of 30cm. The total number of shrubs within each radius is provided. All shrubs were geotagged and given a unique identifying number. Site coordinates are provided.

| **Site Code** | **Semi-arid Region** | **Latitude** | **Longitude** | **Number of Shrubs (n)** | **Percent Cover**  **(%)** |
| --- | --- | --- | --- | --- | --- |
| **Cuyama_1** | San Joaquin | 34.849 | -119.483 | 12 | 14.42 |
| **Cuyama_2** | San Joaquin | 34.854 | -119.486 | 18 | 31.18 |
| **Cuyama_3** | San Joaquin | 34.938 | -119.481 | 21 | 27.98 |
| **Carrizo_3** | San Joaquin | 35.163 | -119.675 | 4 | 21.0 |
| **Carrizo_4** | San Joaquin | 35.116 | -119.621 | 6 | 6.68 |
| **Carrizo_soda_shrub** | San Joaquin | 35.119 | -119.629 | 21 | 8.55 |

1. **Plant List** A list of all observed annual plant species’ Latin binomial names are provided. Mean abundance values for each species are provided at both the shrub and open microsites.

| **Latin Binomial Name** | **Microsite** | **Mean Abundance** |
| --- | --- | --- |
| Acmispon wrangelianus | open | 13.5 |
| Acmispon wrangelianus | shrub | 3.5 |
| Agoseris grandiflora | open | 3.33 |
| Agoseris grandiflora | shrub | 5 |
| Allium peninsulare | open | 40.25 |
| Amsinckia intermedia | open | 33.33 |
| Amsinckia intermedia | shrub | 49.5 |
| Amsinckia tessellata | open | 36.14 |
| Amsinckia tessellata | shrub | 6.57 |
| Astragalus lentiginosus nigricalycis | open | 3 |
| Astragalus lentiginosus nigricalycis | shrub | 1 |
| Brassica nigra | shrub | 1 |
| Bromus madritensis rubens | open | 116 |
| Bromus madritensis rubens | shrub | 81.58 |
| Calandrinia menziesii | open | 75.5 |
| Calandrinia menziesii | shrub | 9 |
| Camissonia strigulosa | open | 6.8 |
| Caulanthus lasiophyllus | open | 2 |
| Caulanthus lasiophyllus | shrub | 6 |
| Eremalche exilis | shrub | 25 |
| Eriastrum densifolium | open | 2 |
| Erodium cicutarium | open | 206.68 |
| Erodium cicutarium | shrub | 13.16 |
| Gutierrezia californica | open | 6 |
| Lactuca serriola | shrub | 1 |
| Lasthenia gracilis | open | 443.54 |
| Lasthenia gracilis | shrub | 4 |
| Lepidium nitidum | open | 72.42 |
| Lupinus microcarpus | open | 1 |
| Lupinus microcarpus | shrub | 1 |
| Monolopia lanceolata | open | 9.69 |
| Monolopia lanceolata | shrub | 2.75 |
| Pectocarya penicillata | open | 114 |
| Pectocarya penicillata | shrub | 10.2 |
| Phacelia tanacetifolia | open | 2 |
| Phacelia tanacetifolia | shrub | 25 |
| Pholistoma membranaceum | shrub | 2 |
| Plagiobothrys arizonicus | open | 36 |
| Poa secunda | open | 10 |
| Schismus barbatus | open | 262.89 |
| Schismus barbatus | shrub | 28.38 |
| Tropidocarpum gracile | open | 10 |

**
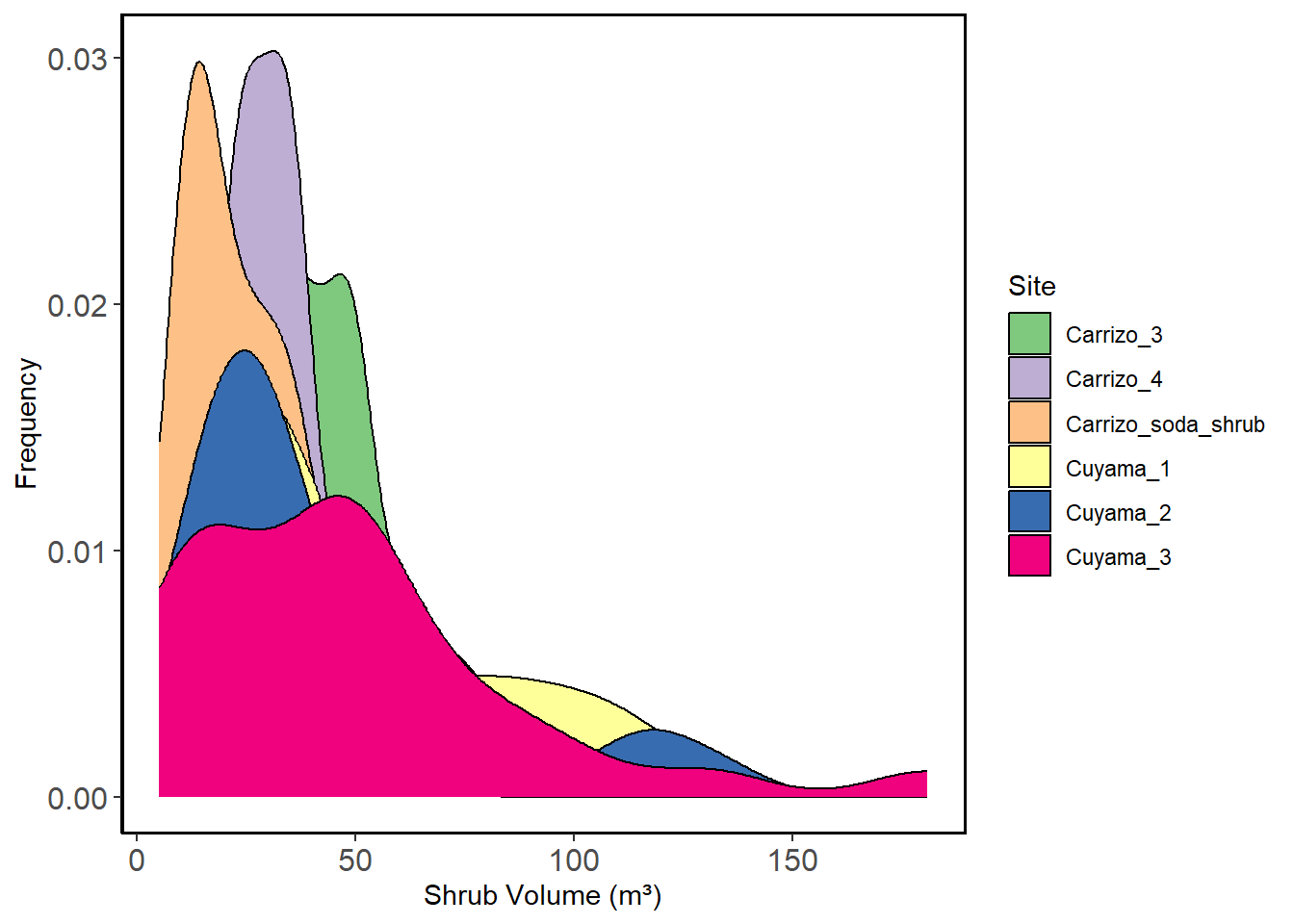
**

1. **Frequency Distribution Density** showing shrub volume (m^3^) at each site on the x-axis. The y-axis represents the frequency at which the particular volume was found. Colour represents the site.

**
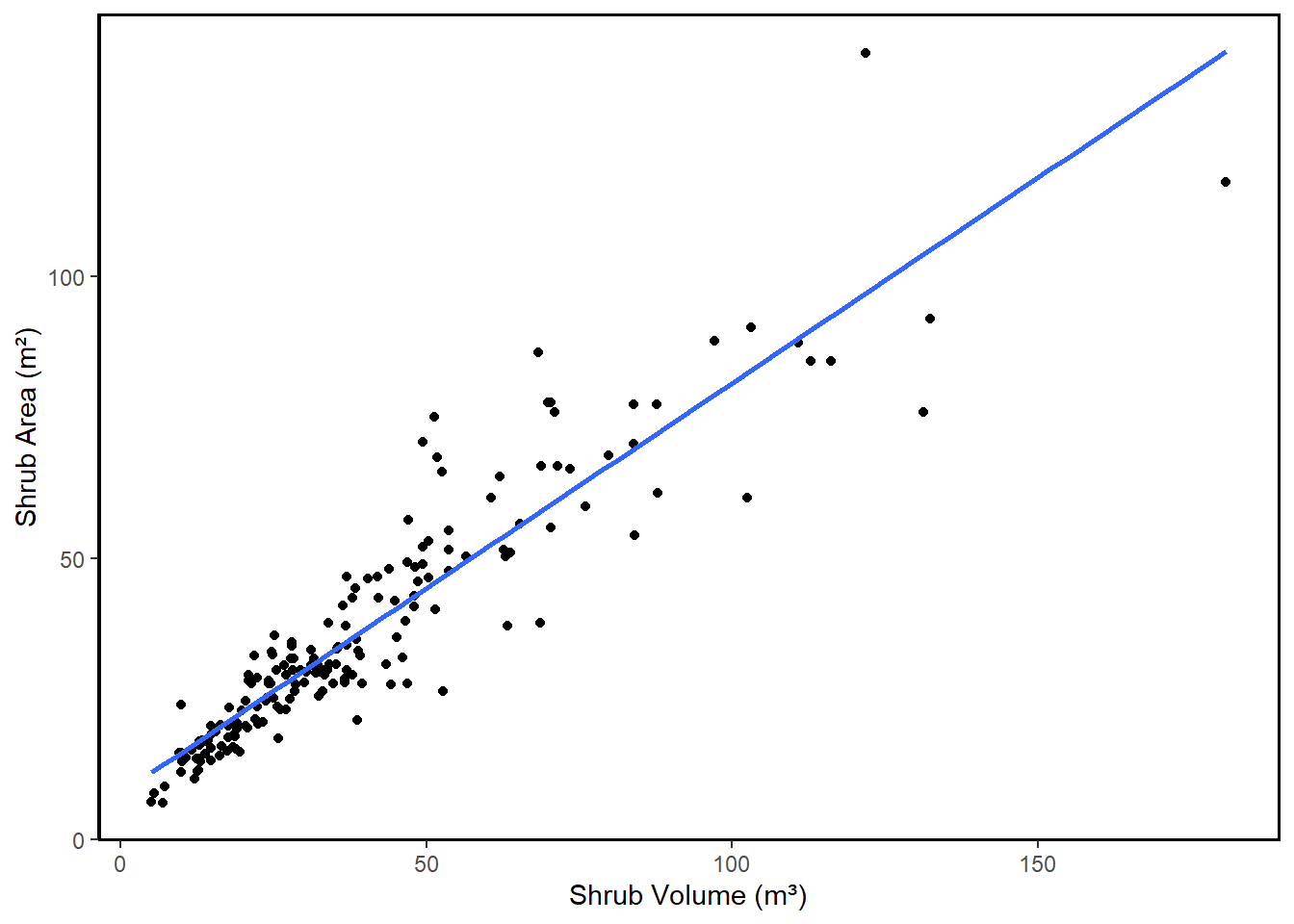
**

1. **Shrub Volume vs. Shrub Area** scatterplot depicting the overall relationship of shrub volume (m^3^) and shrub area (m^2^) (Pearson’s product-moment correlation = 0.918, p<0.001). Blue line represents the smooth conditional mean fitted using the method GLM.

**
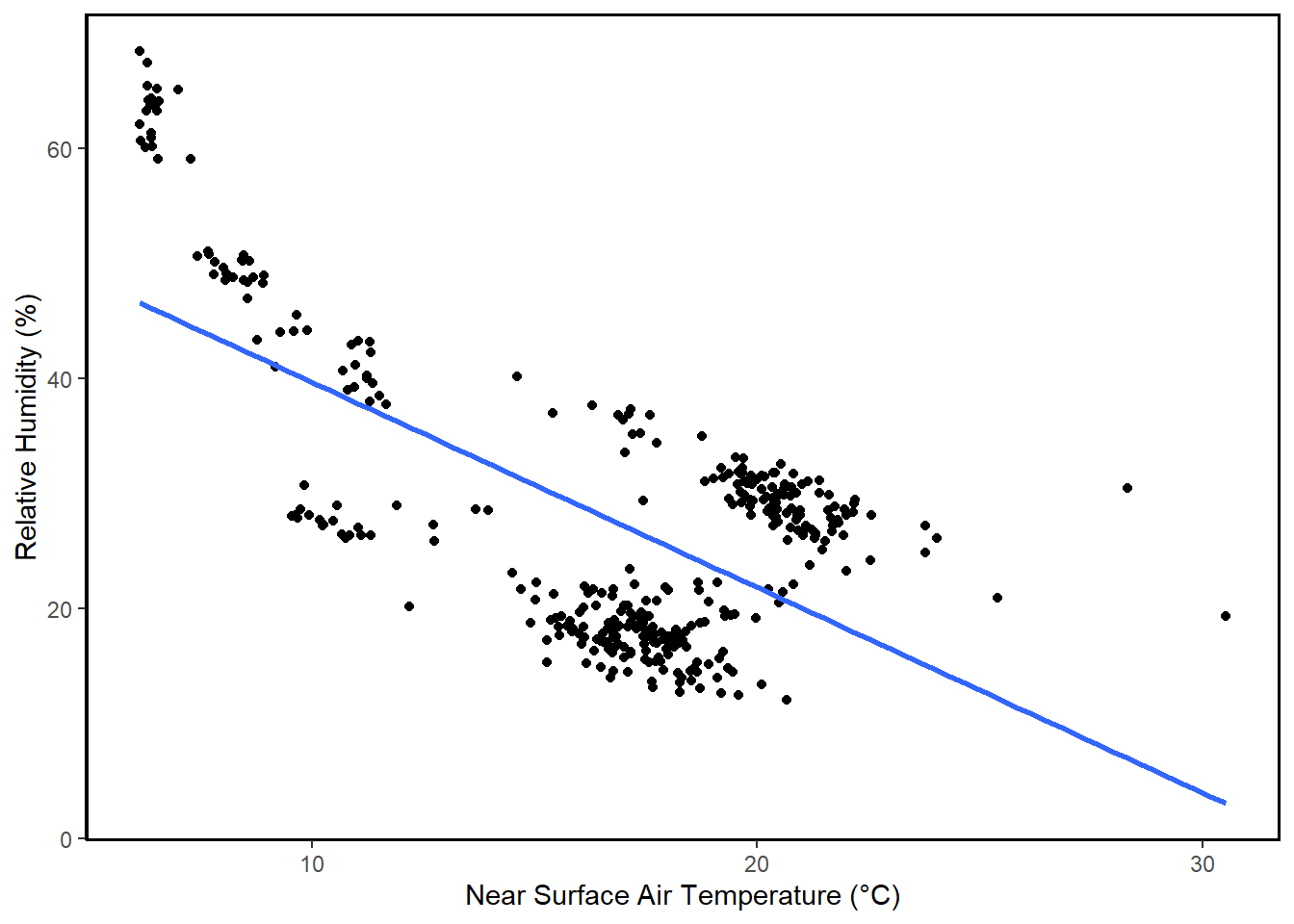
**

1. **NSRH vs. NSAT** scatterplot depicting the overall relationship between near-surface relative humidity (%) and near-surface air temperature (ºC) (Pearson’s product-moment correlation = -0.652, p<0.001). Blue line represents the smooth conditional mean fitted using the method GLM.

**
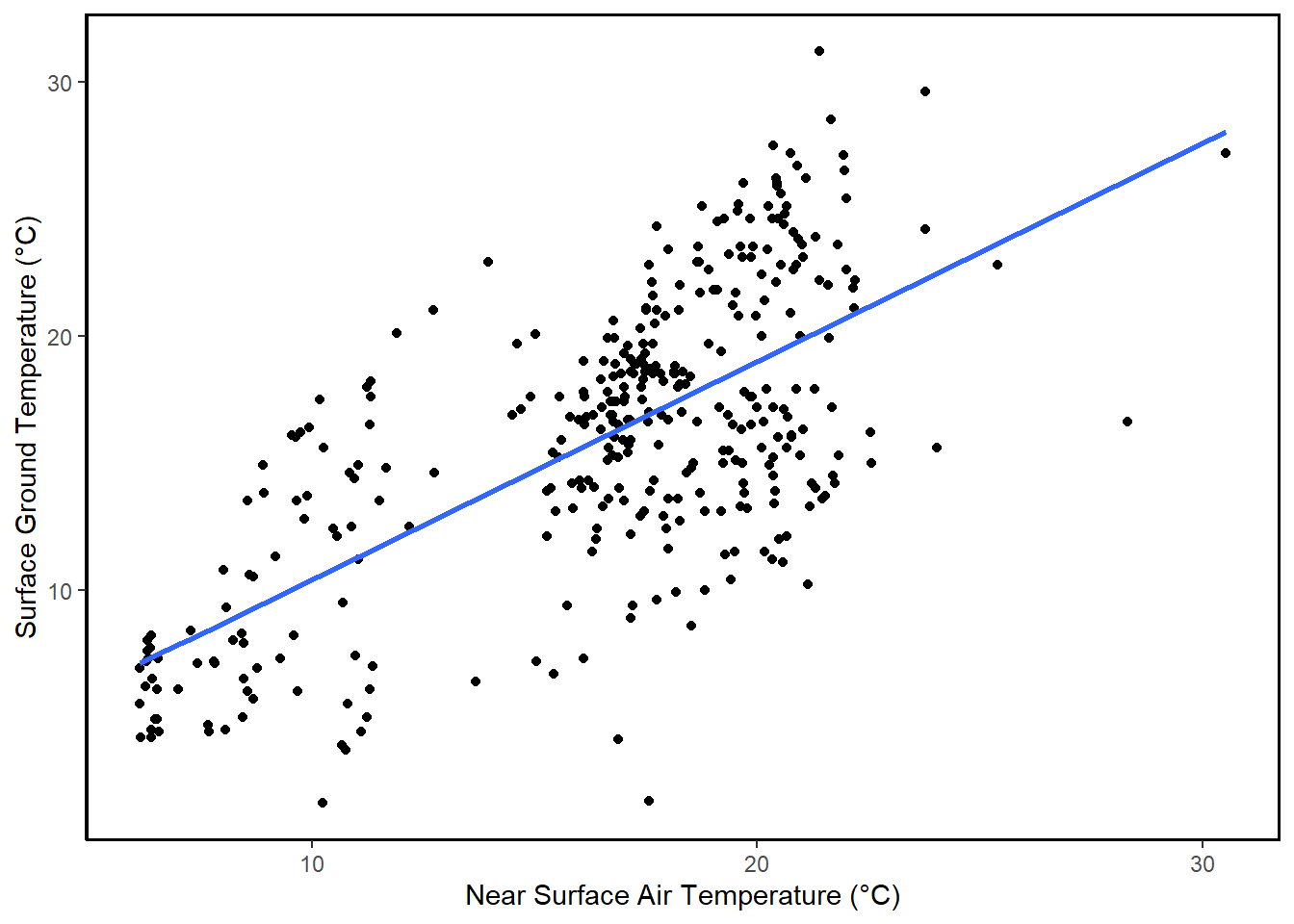
**

1. **SGT vs. NSAT** scatterplot depicting the overall relationship between surface ground temperature (ºC) and near-surface air temperature (ºC) (Pearson’s product-moment correlation = 0.679, p<0.001). Blue line represents the smooth conditional mean fitted using the method GLM.

**
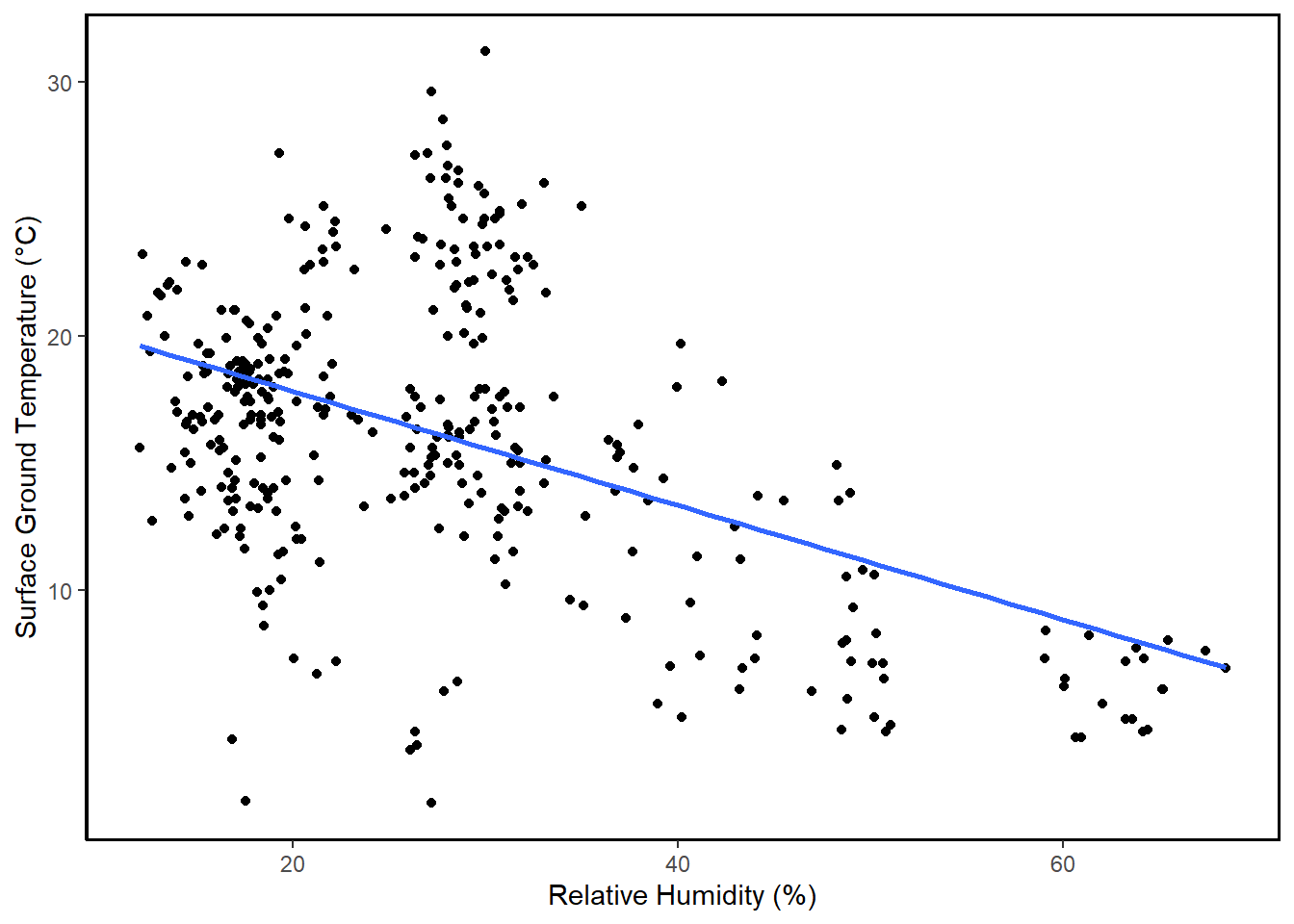
**

1. **SGT vs. NSRH** scatterplot depicting the overall relationship between surface ground temperature (ºC) and near-surface relative humidity (%) (Pearson’s product-moment correlation = -0.487, p<0.001). Blue line represents the smooth conditional mean fitted using the method GLM.

**
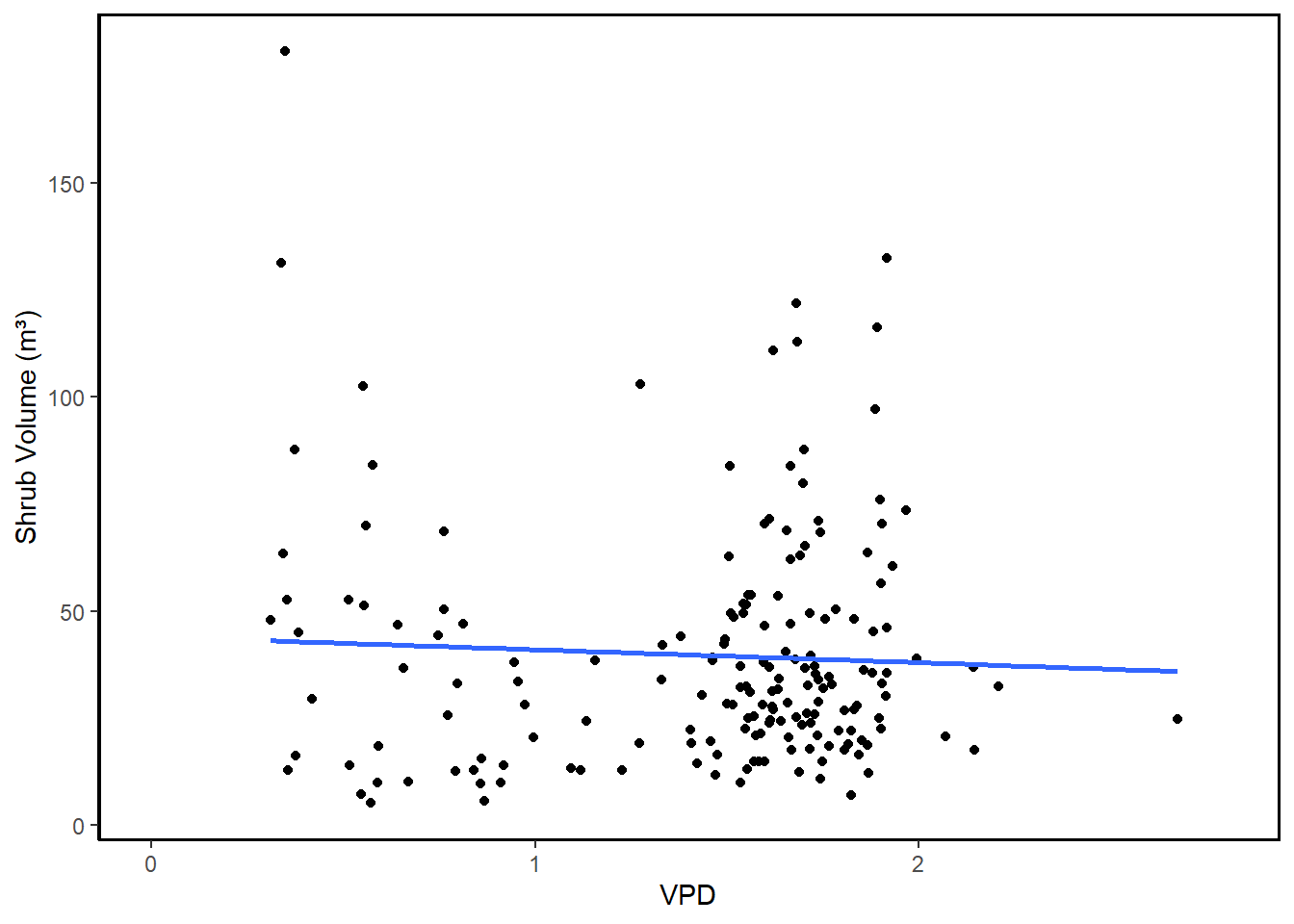
**

1. **Shrub Volume vs VPD** scatterplot depicting the overall relationship between shrub volume (m^3^) and vapour pressure deficit (VPD) (Pearson’s product-moment correlation = -0.0523, p<0.485). Blue line represents the smooth conditional mean fitted using the method GLM.

**
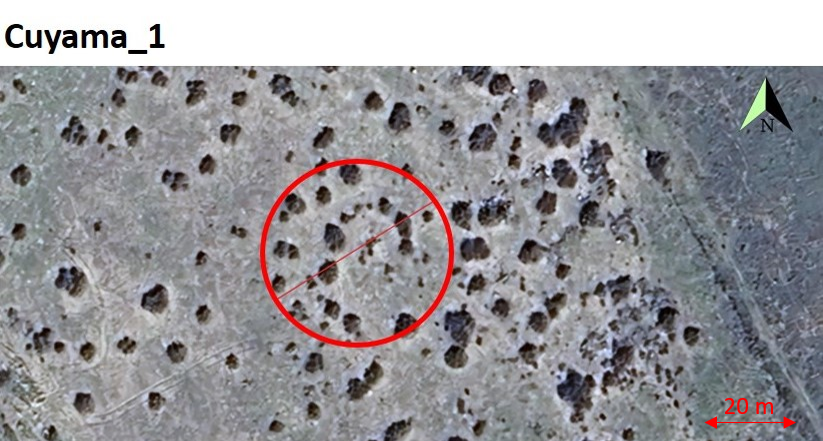
**

**
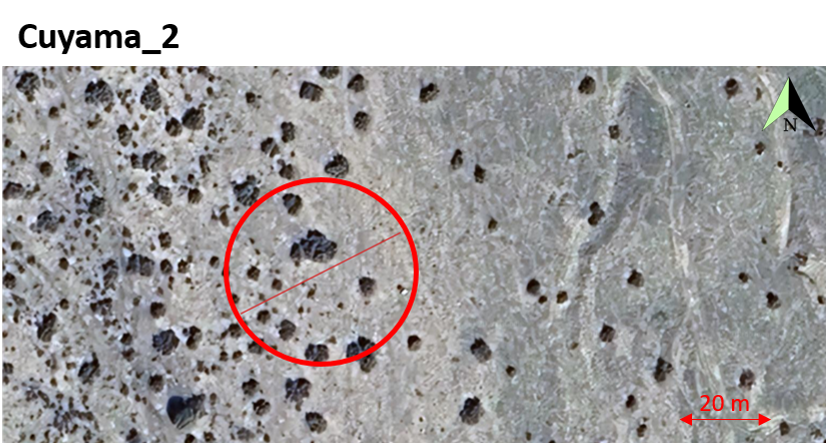
**

**
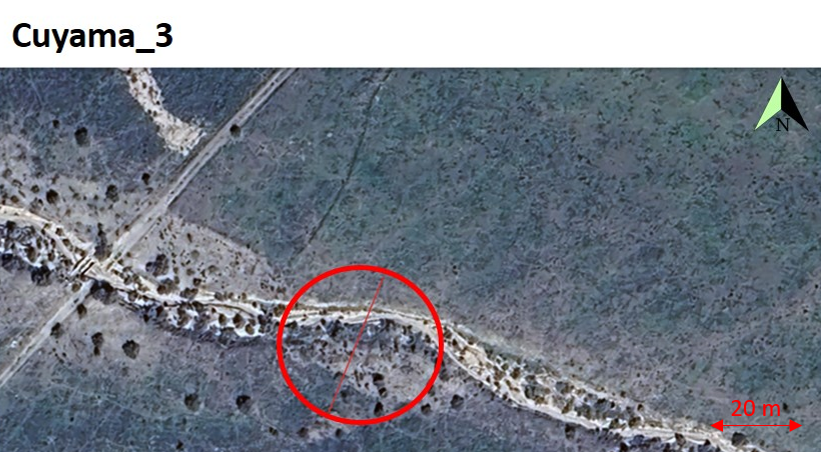
**

**
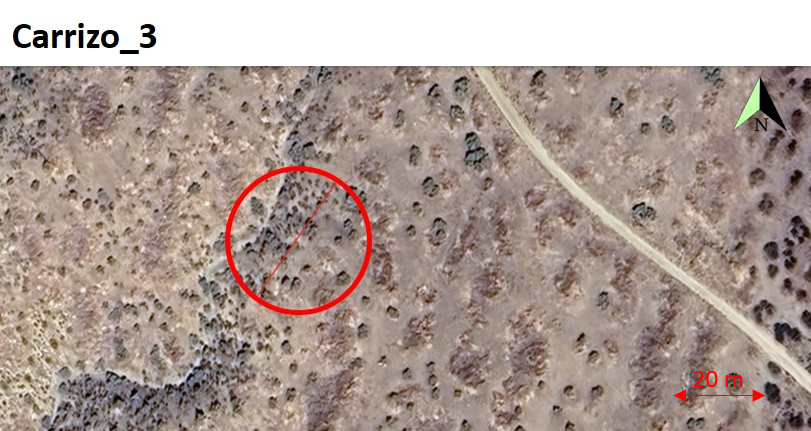
**

**
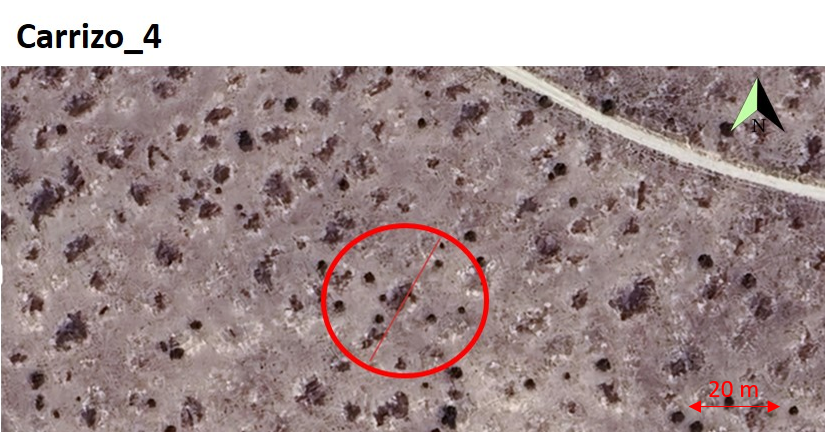
**

**
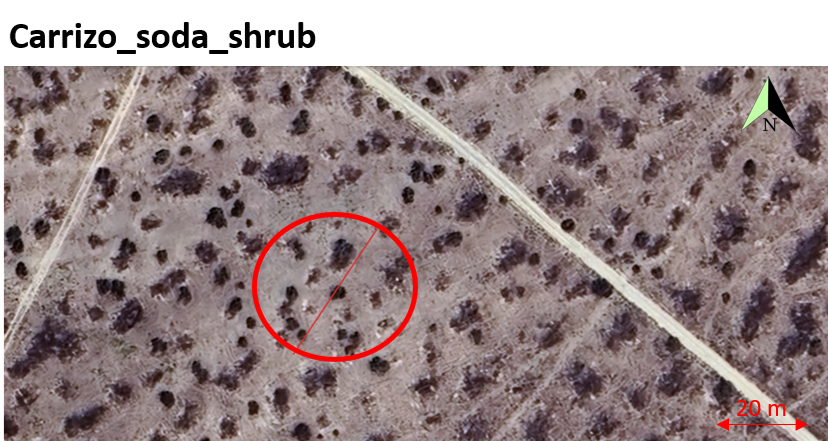
**

1. **Satellite Imagery of Shrub Cover** A circular plot with with a 40m diameter used on *Google Earth* composite satellite images provided by *Airbus* at a spatial resolution of 30cm is shown at each site. The zoom is set the same in each instance.
